# Health system outcomes and determinants amenable to public health in industrialized countries: a pooled, cross-sectional time series analysis

**DOI:** 10.1186/1471-2458-5-81

**Published:** 2005-08-02

**Authors:** Onyebuchi A Arah, Gert P Westert, Diana M Delnoij, Niek S Klazinga

**Affiliations:** 1 Department of Social Medicine, Academic Medical Center, University of Amsterdam, PO Box 22700, 1100 DE Amsterdam, the Netherlands; 2 Netherlands Institute for Health Sciences, Erasmus MC, PO Box 1738, 3000 DR Rotterdam, the Netherlands; 3 Center for Prevention and Health Services Research, National Institute of Public Health and the Environment (RIVM), PO Box 1, 3720 BA Bilthoven, the Netherlands; 4 Tranzo, Faculty of Social and Behavioural Sciences, Tilburg University, PO Box 90153, 5000 LE Tilburg, the Netherlands; 5 Netherlands Institute for Health Services Research (Nivel), PO Box 1568, Utrecht 3500 BN, the Netherlands

## Abstract

**Background:**

Few studies have tried to assess the combined cross-sectional and temporal contributions of a more comprehensive set of amenable factors to population health outcomes for wealthy countries during the last 30 years of the 20^th ^century. We assessed the overall ecological associations between mortality and factors amenable to public health. These amenable factors included addictive and nutritional lifestyle, air quality, public health spending, healthcare coverage, and immunizations.

**Methods:**

We used a pooled cross-sectional, time series analysis with corrected fixed effects regression models in an ecological design involving eighteen member countries of the Organisation for Economic Cooperation and Development during the period 1970 to 1999.

**Results:**

Alcohol, tobacco, and fat consumption, and sometimes, air pollution were significantly associated with higher all-cause mortality and premature death. Immunizations, health care coverage, fruit/vegetable and protein consumption, and collective health expenditure had negative effects on mortality and premature death, even after controlling for the elderly, density of practicing physicians, doctor visits and per capita GDP. However, tobacco, air pollution, and fruit/vegetable intake were sometimes sensitive to adjustments.

**Conclusion:**

Mortality and premature deaths could be improved by focusing on factors that are amenable to public health policies. Tackling these issues should be reflected in the ongoing assessments of health system performance.

## Background

Western populations currently enjoy unprecedented wealth and longevity [1]. It is held that medical care – or more broadly healthcare including public health – contributed immensely to the increased longevity. However, McKeown [2], McKinlay et al [3,4], Illich [5] and others have questioned the role of medical care in these gains. Others, like Mackenbach [6,7] and Bunker et al [8,9] contend that medical care has contributed reasonably to the mortality decline. Unfortunately, these debates have done little to further the course and relevance of health systems today. Resource constraints, poor quality, and the controversial *World Health Report 2000 *[10] have all led to the increased assessment of health systems in terms of effectiveness [11], safety [12], equity and responsiveness [10]. Meanwhile, the functioning of the public health aspect of health systems has received less attention. Functional public health systems are nowadays seen within the context of health promotion and integral social structures [13]. This focus on health promotion strategies that are embedded in societal structures has been described as public health coming "full circle" in the "new public health" era [14].

The new public health hopes to address major risk factors implicated in the global burden of disease. These factors, which threaten the substantial health gains made in the 20^th ^century, include addictive behavior (such as tobacco smoking), nutritional lifestyle (e.g. fat consumption), degrading environmental quality (e.g. air pollution), and less-than-adequate public health investments, coverage and preventive interventions (e.g. immunizations) [15]. Despite the recent increase in studies that either looked primarily at such factors or controlled for them in their analyses [16-22], few studies have tried to assess the combined cross-sectional and temporal contributions of a more comprehensive set of amenable factors to population health outcomes for wealthy countries during the last 30 years of the 20^th ^century.

Therefore, this study uses a pooled, cross-country time-series design to assess the ecological relationships of such amenable factors to population health, adjusting for demographic, national wealth, and medical care-related factors in selected industrialized countries during the period 1970–1999. Population health is a commonly used compound indicator of health system and social performance, and is captured in this study as all-cause mortality and all-cause potential years of life lost. We aim to speak to the relevance of public health-related issues, and their place in assessing health system performance.

### Theoretical framework

Public Health, as an organized effort of society [23], espouses several principles, namely: (a) emphasis on collective responsibility and role of the state; (b) focus on whole populations; (c) emphasis on prevention; (d) concern for the underlying socio-economic determinants of health and disease; (e) multi-disciplinary approach (both quantitatively and qualitatively); and (f) partnerships with populations served [24]. These principles form a useful basis for evaluating the functioning of public health systems [25]. Simply put, medical care (with its emphasis on personal clinical services) and public health (with its emphasis on collective societal efforts for population health) represent the two traditional components of a health system [26].

Given public health's broad focus on population health, the theoretical framework for this study is based on a population health determinants model [27-31]. There are many determinants of population health which are commonly classified as either proximal or distal [18]. The proximal determinants have direct effects on health, and the distal determinants have indirect effects.

The proximal determinants, which act on both micro and macro levels, often include lifestyle or behavior (e.g. alcohol, fat, tobacco, fruit and vegetable consumption), and socioeconomic environment (including macro-economic measures such as wealth), demography (e.g. elderly proportion of the total population), physical environment (e.g. air pollution by oxides of sulphur, nitrogen or carbon) and host constitution. The health system, which also operates at this proximal level, shares an interface with other sectors of organized societies such as the social, political and economic systems. Health system inputs such as physicians and medical technology may be the result of intersectoral dynamics and social choices [32]. It is expected that public health systems can influence many of the proximal non-medical determinants and avert or minimize the need for expensive medical care.

Distal determinants of health include the national, institutional, political, legal, and cultural factors that indirectly influence health by acting on the more proximal factors, their interrelated mechanisms, levels, trends, and distributions. These distal factors are usually more stable than proximal determinants. Though we do not address distal factors in this study, we can roughly capture their potential impact on mortality over time by using dummy variables to account for any unmeasured time-dependent heterogeneity introduced by these distal determinants.

## Methods

We use an ecological design – a pooled cross-sectional, time series analysis of secondary data [33,34] – to quantify the relationship between average population health and factors amenable to public health, taking into account demographic, medical, and macro-economic (that is, crude national wealth) determinants of health. The analysis is based on the country-year units of selected eighteen member countries of the Organisation of Economic Cooperation and Development (OECD) from 1970 to 1999.

### Data and measures

The data for this study are derived from secondary sources [35,36]. The eighteen countries are a convenient sample of wealthy societies whose health experiences are different enough to allow for variation, but similar enough to support effective pooling. These countries, which were used in a recent study on primary care and mortality, are Australia, Belgium, Canada, Denmark, Finland, France, Germany, Greece, Italy, Japan, the Netherlands, Norway, Portugal, Spain, Sweden, Switzerland, the United Kingdom, and the United States [18]. The data management and preparation follow the methods used in that study [18].

The outcome measures of health system performance used are age- and sex-standardized *all-cause total mortality*, and *potential years of life lost *(PYLL) from all-causes. Both types of measures, expressed per 100,000 population, are standardized to the 1980 OECD population. The all-cause PYLL is a measure of premature, but preventable death before age 70 years. PYLL is calculated by summing up deaths occurring at each age and multiplying this with the number of remaining years to live up to the selected 70-year limit. Both all-cause mortality and PYLL have been used in ecological studies of health outcomes [18,37,38].

The independent measures represent four convenient blocks of amenable factors: addictive behavior/lifestyle; nutritional lifestyle; environment, investment and coverage; and disease preventive measures. *Alcohol *(measured in liters per capita) and *tobacco *(in grams per capita) consumption represent the commoner addictive lifestyle factors used in studies [18]. Consumptions of *fat*, *fruit/vegetable *(both in kilograms per capita), and *protein *(measured in grams per capita per day) reflect nutritional lifestyle. The OECD data on nutritional factors come from the FAOSTAT database of the Food and Agriculture Organization of the United Nations [39].

Air pollution is measured as the emission of *nitrogen oxide *in kilograms per capita, and is expected to have negative effects on health and the environment [40]. *Collective (public) health expenditure *is a measure of national expenditure on prevention, public health services and health administration (excluding medical or personal care expenditure), and is hypothesized to have a negative effect on mortality. We quantified collective expenditure as a percentage of GDP to reduce collinearity. Although already at high levels in the selected OECD countries, *healthcare coverage *is a good structural variable for the percentage of the total population that can access public healthcare goods and services included in total public health expenditure, independent of the scope of cost sharing. Here, air pollution, collective health expenditure, and healthcare coverage represent the environment, public health investment and coverage.

Percentages of children reaching their first birthday who were immunized against *measles *and *DTP *(diphtheria, tetanus and pertussis) are used as measures of preventive functions of public health, though they could also serve as outcomes. However, we used two dummy variables to control for 1980s and 1990s fixed effects, and omitted variable bias and to eliminate any unobserved heterogeneity [34]. The 1970s served as reference.

To account for demographic, medical care, and macro-economic factors, we chose several well-documented measures. These were: percentage of the total population aged 65 year and above as a demographic measure (*elderly*); practicing *physician density *per 1,000 population and per capita *doctor visits *as measures of medical care input; and population standardized gross domestic product (*GDP per capita*) as a measure of national wealth and a proxy for medical expenditures [18]. We deflated the GDP per capita by using the constant 1995 US dollar.

### Design and analysis

We employed a pooled cross-sectional time series design that entailed stacking the eighteen countries (also referred to as cross-sections) over time. This resulted in a combination of cross-sections and time series with a matrix configuration that considered variation between cross-sections before variation within cross-sections over time. The obvious advantage of using a pooled ecological design is that it increases statistical power. We used fixed effects regression models and robust statistical modeling techniques to overcome repeated measure biases, correlated errors and heterogeneity [41-44]. The final regression model is specified as follows:

*Y*_*nt *_= *γ*_*t *_+ *β*_*0 *_+ *β*_*k*_*X*_*knt *_+ *ε*_*nt*_

where

*n *= 1....18 countries

*t *= 1....*T *time points (calendar years from 1970 through 1999)

*k *= 1....*K *number of independent variables

*γ*_*t *_= set of time effect dummy variable(s)

*β*_*0 *_= constant

*β*_*k *_= pooled regression estimates of the effect of each independent variable

*X*_*knt *_= independent variables per country for each unit year

Unfortunately, simply because the above regression equation includes both stochastic and non-stochastic variables, the expected value of the error term is not zero and the variance is not constant. By assuming all the coefficients (the *β*_*k*_'s) to be the same for each cross-section within the regression model, we compound the problems of heteroscedasticity (that is, non-constant variances) or autoregression (that is, decaying variance due to correlated error terms over time). Autoregression can only be addressed after heteroscedasticity has been corrected for, since both anomalies cannot be visualized at the same time [33]. For pooled data with correlated errors, the ordinary least squares (OLS) method does not yield the correct standard errors on which to base the hypothesis or relationship testing under study. To correct for heteroscedasticity in this sample, we employ heteroscedasticity-consistent standard error (HCSE) estimators which use the square of the residuals from the OLS equation to approximate the variance-covariance structure of the regression estimates [41-44]. Since most of these estimators have not been fully implemented in routine statistical software packages, we use a special HCSE macro to model the heteroscedasticity-consistent covariance matrix [44]. Autoregression is then addressed via a quasi-differencing technique [34].

Alternatively, the pooled cross-sectional time series data could be treated as repeated observations on each cross-section, although strictly speaking, here, they represent average national characteristics per unit time [34]. Similar results are obtained for this study by using the Linear Mixed Models procedure of SPSS (version 12.0.2, SPSS Inc., IL, March 2004) and the SAS PROC MIXED (version 8.2, SAS Institute Inc, NC, 2001) procedure to model the means, variances and covariances of this data as repeated measures. However, these general softwares implement a heteroskedastic-consistent matrix method which uses conditional variance of the error, rather than the more robust methods which we used to adjust each squared residual by a function of how deviant the pattern of independent variables of each cross-section is [43].

We used five pre-specified nested models to address the question 'after adjusting for demographic, medical care input, and wealth, can mortality and premature death be still explained by factors amenable to public health?' The first four models used measures of addictive behavior/lifestyle; nutritional lifestyle; environment, investment and coverage; and preventive measures. Model 1 examined the effects of tobacco and alcohol on mortality. Model 2 extended model 1 to include fat, fruit/vegetable and protein consumption. Model 3 added air pollution, collective health expenditure, and health care coverage. In model 4, we included measles and DTP immunizations. Finally, model 5 further adjusted for time fixed effects using per decade time dummies with the first decade (1970s) as reference [34]. We also re-estimated models 3 to 5 after excluding the United States from the healthcare coverage variable, given the known lower levels of healthcare coverage in the American population. In addition, we re-ran these models excluding the healthcare coverage variable entirely in order to gauge its impact on the models considering that healthcare coverage also reflects access to care in general, not just public health services. We present statistics for hypothesis testing, model improvement and the proportion of total variance (as adjusted R squared) in outcomes explained in the final models, along with the regression estimates and their modeled standard errors.

## Results

Between 1970 and 1999, all-cause mortality and PYLL decreased on the average by approximately 27% and 37% respectively across the selected countries (Table 1). Protein and fruit/vegetable consumption increased while alcohol, tobacco, and fat intake decreased, albeit with substantial variation across countries. Fat intake actually increased in Greece, Italy, Japan, Portugal and Spain by about 0.2 to 2% annually [35]. Immunization levels and collective health expenditure also increased. The healthcare coverage levels improved by about 2 to 4 percentage points when the United States was excluded from the pool. There were also substantial increases in the elderly population, national wealth, density of practicing physicians, and doctor visits during the 1970–1999 period.

**Table 1:** Descriptive statistics for variables used in the pooled cross-sectional time series analysis

**Variables**	**1970–79**		**1980–89**		**1990–99**	
	
	Mean	Standard deviation	Mean	Standard deviation	Mean	Standard deviation
All-cause mortality (both sexes per 100,000 population)	938.25	113.74	789.11	79.79	687.65	83.87
Potential years of life lost (before age 70 years per 100,000 population)	7009.15	1449.67	5372.07	891.38	4424.51	798.37
Tobacco (grams per capita)	2,699.88	450.32	2411.38	455.98	2052.00	502.29
Alcohol consumption (liters per capita)	11.62	3.52	11.33	2.94	9.98	2.27
Fat consumption (kilograms of butter per capita per year)	5.42	3.76	4.90	3.30	3.56	2.42
Fruit & vegetable consumption (kilograms per capita per year)	184.69	61.97	200.14	68.71	220.75	71.03
Protein consumption (grams per capita per day)	93.48	8.03	98.62	7.81	103.06	7.52
Pollution (Nitrogen oxide emission, in kilograms per capita)	41.50	16.29	43.77	20.72	44.71	26.59
Collective health expenditure (% GDP)	0.45	0.24	0.50	0.25	0.53	0.32
Healthcare coverage (% population) [without USA]	90.49 [92.2]	13.69 [12.10]	94.16 [96.75]	13.40 [7.10]	93.84 [97.86]	17.61 [5.96]
DTP immunization (% children)	86.61	12.38	87.09	10.56	91.09	8.71
Measles immunization (% children)	59.23	9.57	72.67	12.07	86.58	11.19
Elderly (percentage of population over 65 years)	11.58	2.09	13.09	1.93	14.73	1.64
Physician density (per 1,000 population)	1.58	0.32	2.29	0.45	2.87	0.58
Doctor visits (per capita)	4.08	1.19	5.39	2.17	6.2	2.55
GDP per capita (in constant 1995 US dollars)	17,750.94	7,029.03	21,543.59	8,110.65	25,783.31	9,306.90

Table 2 details the regression results for all-cause mortality. Here, tobacco was positively associated with all-cause mortality in models 1 to 3, but was not significant in models 4 and 5 that included immunization variables and time dummies. Alcohol was significantly and positively associated with mortality in all the models, yielding mortality increases of 6.6 to 8.6 per 100,000 populations for every one-liter increase in per capita alcohol consumption. Fat consumption also showed a strong positive relationship to mortality in all cases. Fruit/vegetable consumption was only negatively related to mortality in the full model and when the model excluded environmental, collective health spending, coverage and immunizations. Protein showed a stable negative association in all models, changing only slightly in model 3. Air pollution was hardly related to mortality in all models that included healthcare coverage. Collective health expenditure, healthcare coverage, and immunizations were all negatively associated with higher mortality (*P *< 0.01).

**Table 2:** Regression estimates from the pooled cross-sectional time series analysis of all-cause mortality per 100,000 population

**Variables**	**Model 1 **Estimate (S.E.)	**Model 2 **Estimate (S.E.)	**Model 3 **Estimate (S.E.)	**Model 4 **Estimate (S.E.)	**Model 5 **Estimate (S.E.)
Constant	949.32*** (45.75)	1,256.36*** (64.55)	1,487.51*** (80.04)	1,548.45*** (91.38)	1,491.87*** (93.78)
Tobacco	0.03*** (0.01)	0.03*** (0.01)	0.02** (0.01)	0.01 (0.01)	0.01 (0.01)
Alcohol	8.59*** (1.49)	8.59*** (1.50)	6.56*** (1.46)	8.03*** (1.46)	8.61*** (1.52)
Fat consumption		9.44*** (1.66)	9.83*** (1.91)	9.83*** (1.91)	8.46*** (1.96)
Fruit/vegetable consumption		-0.20* (0.08)	-0.03 (0.09)	-0.10 (0.09)	-0.19* (0.09)
Protein consumption		-3.22*** (0.57)	-4.25*** (0.70)	-3.93*** (0.76)	-3.52*** (0.72)
Air Pollution			0.13 (0.20)	0.46 (0.25) *P *= 0.07	0.48 (0.26) *P *= 0.06
Collective health expenditure			-72.44*** (18.02)	-68.98*** (18.08)	-48.95* (19.13)
Healthcare coverage			-1.89*** (0.30)	-1.73*** (0.31)	-1.44*** (0.32)
DTP immunization				-1.39*** (0.31)	-1.39*** (0.31)
Measles immunization				-0.88** (0.27)	-0.88** (0.27)
Elderly	4.55* (2.26)	1.47 (2.04)	0.60 (2.19)	4.83* (2.19)	4.87* (2.12)
Physician density	-103.65*** (6.34)	-47.81*** (7.52)	-49.04*** (8.07)	-20.57* (8.07)	-3.92 (8.74)
Doctor visits	-15.55*** (1.75)	-9.57*** (1.70)	-4.90** (1.80)	-5.31** (1.71)	-3.60* (1.68)
GDP per capita	-0.003*** (0.0004)	-0.006*** (0.0005)	-0.006*** (0.0005)	-0.005*** (0.0005)	-0.005*** (0.0005)
1980s fixed effects^++^					-60.78*** (9.93)
1990s fixed effects^++^					-83.04*** (14.11)
**F change**	-	38.44^#^	15.03^#^	26.57^#^	17.32^#^
**Adjusted R^2^**	0.57	0.66	0.79	0.72	0.74

S.E.: standard error of estimate; *P < 0.05; **P < 0.01; ***P < 0.001; ^++^decade time effects to eliminate omitted-variable bias due to unobserved heterogeneity with reference to 1970s baseline; ^#^significantly better than preceding model and model 1 (*P < 0.000*); n = 451

Exclusion of the United States data on healthcare coverage did not substantially alter the models, nor did change its association with mortality. Although the total exclusion of the healthcare coverage variable did not improve the fit of the models (not reported in Table 2), it significantly changed the mortality associations of tobacco, fruit/vegetable consumption, and air pollution. Regression coefficients for tobacco changed from 0.02 to 0.06 (*P *< 0.001) and 0.01 to 0.02 (*P *< 0.0) in only models 3 and 4 respectively. For fruit/vegetable consumption, the estimates changed from -0.03 to -0.37 (*P *< 0.001) and -0.10 to -0.25 (*P *< 0.01) in only models 3 and 4 respectively. For air pollution, the estimates changed from 0.13 to 1.40 (*P *< 0.001), 0.46 to 0.79 (*P *< 0.01) and 0.48 to 0.74 (*P *< 0.01) in models 3, 4 and 5 respectively. GDP per capita remained strongly negatively associated with mortality in all models. Except for the elderly proportion, all medical input adjustment covariates were significantly associated with lower mortality. The total explained variance in all-cause mortality ranged from 57% in model 1 to 74% in the fully adjusted model 5.

Table 3 summarizes the corrected fixed effects regression results for age- and sex-standardized all-cause PYLL, a measure of premature death. Five stepwise contemporaneous adjusted models are presented, with each model compared to the preceding one and model 1 for improvements. Again, tobacco was significantly associated with higher PYLL in all models except those corrected for immunization and time effects. Alcohol and fat intake were positively related to higher PYLL. Fruit/vegetable intake was only negatively associated with PYLL in model 3 which did not correct for immunization and time effects. Air pollution tended to be significantly and positively associated with premature death in the fuller models (*P *< 0.001). Protein, collective health expenditure, healthcare coverage, and immunizations all strongly accounted for lower PYLL. As for total all-cause mortality, excluding the United States data on healthcare coverage did not substantially alter our estimates for factors associated with PYLL. A total exclusion of the healthcare coverage variable improved the regression coefficients of air pollution from 2.92 to 20.14 (*P *< 0.001) in model 3, and fruit/vegetable consumption from -0.21 to -2.21 (P < 0.05) in model 5 only. For the premature death outcome, the regression coefficients varied more in strength than was seen in Table 2 for all-cause mortality. The full, time-adjusted model accounted for 79% of the total variance in PYLL in the pooled countries.

**Table 3:** Regression estimates from the pooled cross-sectional time series analysis of all-cause potential years of life lost per 100,000 population

**Variables**	**Model 1 **Estimate (S.E.)	**Model 2 **Estimate (S.E.)	**Model 3 **Estimate (S.E.)	**Model 4 **Estimate (S.E.)	**Model 5 **Estimate (S.E.)
Constant	7,611.05*** (489.65)	9,020.45*** (831.22)	13,168.22*** (1,221.73)	13,906*** (869.99)	13,458.57*** (890.95)
Tobacco	0.27** (0.10)	0.25* (0.10)	0.14* (0.07)	0.01 (0.07)	0.12 (0.07) *P *= 0.09
Alcohol	122.74*** (17.18)	120.99*** (17.65)	87.09*** (17.94)	104.86*** (13.51)	110.26*** (13.59)
Fat consumption		67.99*** (17.19)	142.39*** (15.96)	102.44*** (15.20)	91.90*** (15.05)
Fruit/vegetable consumption		-0.61 (0.85)	-3.08** (1.07)	-1.09 (0.85)	-0.21 (0.84)
Protein consumption		-14.97* (6.46)	-31.04*** (7.02)	-22.59*** (6.62)	-19.01** (6.38)
Air Pollution			2.92 (1.8)	7.31*** (2.16)	7.57** (2.30)
Collective health expenditure			-1,268.53*** (261.88)	-1,180.31*** (162.92)	-1,002.32*** (169.98)
Healthcare coverage			-36.32*** (5.32)	-28.31*** (3.24)	-25.37*** (3.30)
DTP immunization				-21.97*** (3.79)	-26.20*** (3.77)
Measles immunization				-21.28*** (2.87)	-14.53** (2.92)
Elderly	-66.14** (23.54)	-103.99*** (20.32)	-70.40*** (19.75)	-44.80* (18.88)	-47.50** (16.73)
Physician density	-768.98*** (72.85)	-490.75*** (88.92)	-507.20*** (87.45)	-41.25 (79.42)	-193.32* (89.77)
Doctor visits	-129.19*** (17.28)	-92.20*** (14.28)	-10.50 (18.21)	-30.13* (14.25)	-13.71 (14.28)
GDP per capita	-0.035*** (0.005)	-0.052*** (0.006)	-0.053*** (0.005)	-0.039*** (0.005)	-0.04*** (0.005)
1980s fixed effects^++^					-656.13*** (104.82)
1990s fixed effects^++^					-775.84*** (141.07)
**F change**	-	14.02^#^	36.97^#^	71.43^#^	21.89^#^
**Adjusted R^2^**	0.57	0.60	0.69	0.77	0.79

S.E.: standard error of estimate; * *P < 0.05*; ** *P < 0.01*; *** *P < 0.001*; ^++^decade time effects to eliminate omitted-variable bias due to unobserved heterogeneity with reference to 1970s baseline; ^#^significantly better than preceding model and model 1 (*P < 0.000*); n = 451

## Discussion

Our contemporaneous, pooled cross-sectional series analysis suggests that a number of factors that may be amenable to public health had important associations with mortality and premature death in the selected OECD countries during the period 1970 to 1999. Most of these associations were still significant even after controlling for demographics, physician density, visits to the doctor, and GDP per capita. Tobacco, alcohol and fat intake were all positively associated with overall mortality and premature death. Protein consumption, collective health expenditure, healthcare coverage, and immunizations exhibited negative associations with both outcome measures. Though air pollution did not have significant effects on overall mortality, it was sometimes related to higher premature death when other covariates were fully taken into account. Fruit/vegetable consumption showed weak and inconsistent negative effects on both outcome measures, when partially adjusted for other covariates.

Previous studies have showed that some of the factors we studied were important in explaining mortality in OECD countries [12,18,20-22,45]. Though tobacco is known to be strongly related to higher mortality, our results support other findings that tobacco becomes insignificant in fuller models, perhaps suggesting that the tobacco consumption variable is poorly defined in the OECD dataset [18,45]. It has been suggested that a better definition would be "percent of population that smokes every day," but not the per capita use of tobacco we analyzed in this study [18]. Interestingly, in unreported lagged analyses, tobacco only became significant in fully adjusted models with at least 5 year-lags. It is, however, unclear what this might mean or how to determine what an appropriate lag period would be for all covariates in the models. We also found that tobacco was sensitive to adjustments for healthcare coverage and time effects in the total all-cause mortality models, suggesting, perhaps, that while tobacco consumption variable was limited to specific populations within countries, the healthcare coverage variable had a wider reach across populations, effectively diluting the statistical effect of tobacco. Similarly, the failure of fruit/vegetable consumption and air pollution to show the expected associations [15,16] in the fuller models in this study may be due to poor definitions and data quality, or due to their sensitivity to the effect of healthcare coverage in the models.

In its 2002 World Health Report, the World Health Organization showed that lifestyle, behavioral, and environmental risk factors, such as the ones in this study, accounted for significant proportions of the disease and mortality burden in most parts of the world including the affluent countries [15]. As much as 39–40% of the disease burden and 51–53% of mortality in developed countries were attributable to 20 selected risk factors such as tobacco, alcohol, high blood pressure, high body mass index, high cholesterol and low fruit and vegetable intake [17]. Our study is the first, however, to report a pooled time series impact of tobacco, alcohol, fat, fruit/vegetable, air pollution, collective health expenditure, healthcare coverage and immunizations on mortality and premature death, adjusting for demographics, medical care input and national wealth in the selected OECD countries during the last 30 years of the 20^th ^century. It indirectly supports some of the aforementioned population risks, but raises questions as to what public health can actually do to curb the unhealthy associations.

Revisiting the essential functions of public health [24,46,47] and its classical paradigms of health promotion, health protection and disease prevention [14] may offer broad insights into holistic approaches for addressing the effects of health determinants. For instance, nutritional lifestyle factors are amenable to *health promotion *[15]. Air pollution could be addressed under *health protection *activities such as environmental modification and regulations. Immunizations against infections such as measles, diphtheria, tetanus and pertussis belong to the disease *prevention *role of public health. Although, this study is ecological in nature, uses population average variables [48,49], and recognizes the potential for the ecological fallacy [50], it is unlikely that the solutions to the problems of, say, alcohol, tobacco, and nutritional lifestyle would be entirely ecological. Solutions, such as behavioral modification, targeted at all levels of the society, from individuals to groups, would be necessary.

There is evidence that some countries such as the US [51,52] and the Netherlands [53] have ongoing initiatives aimed at tackling health determinants in their populations. It is yet to be seen how successful these programs would be if specific attention is not given to re-engineering public health systems. Increasing healthcare coverage is yet another important way of improving population health and its distribution. This is particularly important for the US where coverage is still a big problem.

Furthermore, the functions of health status monitoring, surveillance, reducing disaster impact, human resource development and public health regulation require substantial investment. Public health investment may have increased relative to GDP in many OECD countries, but the attained levels and distribution of collective health expenditure are still inadequate, given the problems of re-emerging infections, unsolved issues of poverty and inequalities, global terrorism and environmental degradation [54]. Currently, many OECD countries spend far more on the curative medical care sector [55,56] than on prevention and health promotion [35,36]. Unfortunately, many of the diseases (e.g. coronary heart disease) treated in their hospitals, for example, tend to arise from such preventable factors as excessive tobacco, fat and alcohol use [15-17]. Our study showed that even after adjusting for medical care input, there were excess mortality and premature deaths due to preventable factors.

It, therefore, seems prudent to re-focus on public health functions of health systems for at least four reasons. First, it averts health problems and minimizes subsequent morbidity and mortality. Second, public health faces a legitimacy or relevance problem when it does not deal competently with the conflict between civil liberties and health promotion [13], as well as with the new 'epidemics' such as obesity [14,57]. Third, the recent attention given to health system performance should be more comprehensive and include the optimal functioning of public health systems alongside medical care structures [10,11,30,58]. Fortunately, the US, UK, Netherlands, Australia and Canada are among the countries actively pursuing systematic evaluations of their health systems. Fourth, public policy on health and health-related social issues needs to become more integrated, and public health offers an important interface between the traditional health sector and the social sectors. There is need for integrated, intersectoral and innovative solutions beyond the prevailing narrow policy approaches [57,59]. In the light of a similar OECD study that showed that primary care had strong relationship with health outcomes [18], even after controlling for similar factors as we studied, it seems that strengthening primary care and public health may be a prudent and an effective strategy against unfavorable health outcomes. Our study further reinforces recent analyses which used the concept of 'avoidable' mortality (that is, mortality that should not occur in the presence of effective and timely healthcare) to point out the importance of appropriate public health policies as an integral part of evaluating and improving health system performance [60,61].

### Limitations of this study

This study used data that may have comparability and definitional deficiencies [20,35,36]. Use of secondary data from international resources can import the attendant problems of incomparable definitions and poor data quality. The OECD Health dataset (from where we took our public health and medical care related variables) and the OECD's Annual National Accounts data (that provided the expenditure variables in our study) are no exceptions. There are likely issues of errors of observation and comparability in this database given the daunting tasks that underlie such international data collection efforts. The incomparability issues are even more likely to be more severe as the dataset tries to include more non-healthcare accounts measures such as lifestyle factors, as has been the case in recent years. Yet, one can be too apologetic about measurement errors in the OECD Health dataset given its seeming robustness for routine and political use and for guiding practical decisions so far [62]. Besides, efforts are constantly being made to increase the value and quality of the data.

The measures we used are, at best, weak proxies for more robust measures of aggregate lifestyle, environmental quality and safety, public health investment and medical care inputs [61,63]. Medical care input data tend to show mixed results, especially within the context of avoidable mortality [61,64]. Furthermore, this study does not provide clear directions as to which policies are best suited for addressing lifestyle, environment, public health investments or any of the factors we studied. The pooled nature of the statistical models limit the potential for generalizability of our findings to other countries not included in this study. Moreover, the estimated models used crude measures, ignored distributional concerns and distal determinants of health, and did not consider the possible multilevel and/or lagged nature of the explored relationships.

## Conclusion

We have presented a pooled, cross-sectional time series analysis of the associations of public health interventions and investment, the environment, and lifestyle-related factors with population health in selected industrialized countries during the period 1970–1999. Given the limitations of the study, we only make broad-brush assessments of the relevance of these findings. In view of current health concerns, our findings serve to make a case for a "new public health" as a cornerstone of health systems. As such, health policies aimed at preventable factors, namely those modifiable by public health, should count in the overall assessment of health systems.

## Competing interests

The author(s) declare that they have no competing interests.

## Authors' contributions

OAA conceived of the study, collected the data, and led the analysis, interpretation and manuscript drafting. GPW assisted with the study design and strategy for analysis, interpretation of findings and critical review of the manuscript for intellectual content. DMD also assisted with the study design, interpretation of findings and critical review of the manuscript for intellectual content. NSK assisted with the study design, interpretation, and critical review of the manuscript. All authors reviewed the manuscript for intellectual content.

## Pre-publication history

The pre-publication history for this paper can be accessed here:


